# *N*-Acetyldopamine dimers from *Oxya chinensis sinuosa* attenuates lipopolysaccharides induced inflammation and inhibits cathepsin C activity

**DOI:** 10.1016/j.csbj.2022.02.011

**Published:** 2022-02-15

**Authors:** Ashutosh Bahuguna, Tejinder Pal Khaket, Vivek K. Bajpai, Shruti Shukla, InWha Park, MinKyun Na, Yun Suk Huh, Young-Kyu Han, Sun Chul Kang, Myunghee Kim

**Affiliations:** a Department of Food Science and Technology, Yeungnam University, Gyeongsan-si, Gyeongsangbuk-do 38541, Republic of Korea; b Department of Biotechnology, Daegu University, Gyeongsan-si, Gyeongsangbuk-do 78453, Republic of Korea; c Department of Energy and Materials Engineering, Dongguk University-Seoul, Seoul 04620, Republic of Korea; d TERI–Deakin Nanobiotechnology Centre, The Energy and Resources Institute, Gwal Pahari, Gurugram, Haryana 122003, India; e College of Pharmacy, Chungnam National University, Daejeon 34134, Republic of Korea; f Department of Biological Engineering, NanoBio High-Tech Materials Research Center, Inha University, Incheon 22212, Republic of Korea; g Institute of Cell Culture, Yeungnam University, Gyeongsan-si, Gyeongsangbuk-do 38541, Republic of Korea

**Keywords:** Cathepsin C, Essential dynamics, Inflammation, *In silico*, NF-κB, *Oxya chinensis sinuosa*

## Abstract

*Oxya chinensis sinuosa* (rice field grasshopper) is an edible insect with numerous health beneficial properties, traditionally being used to treat many ailments in Korea and other countries. *O. chinensis sinuosa* has been used from centuries, however, a little is known about the chemical functionality of its bioactive compounds. Therefore, this study examined the anti-inflammatory and cathepsin C inhibitory activities of *N*-acetyldopamine dimer (2R, 3S)-2-(3′,4′-dihydroxyphenyl)-3-acetylamino-7-(*N*-acetyl-2″-aminoethyl)-1,4-benzodioxane (DAB1) isolated from *O. chinensis sinuosa*. Results showed that DAB1 reduced the expression of pro-inflammatory mediator (iNOS, COX-2) and cytokines (TNF-α, IL-1β, and IL-6), and curtailed the nuclear translocation of NF-κB by inhibiting the phosphorylation of IκBα in lipopolysaccharide stimulated macrophages. Additionally, DAB1 inhibited cathepsin C activity at the cellular level, supported by *in vitro* assay (Ki, 71.56 ± 10.21 µM and Kis, 133.55 ± 18.2 µM). Moreover, combinatorial molecular simulation and binding free energy analysis suggested a significant stability and binding affinity of cathepsin C-DAB1 complex via formation of hydrogen bond and hydrophobic interactions with the catalytic residues (Gln228, Thr379, Asn380, and Hie381). Also, essential dynamics analysis showed DAB1 induced non-functional motions in cathepsin C structure. Collectively, DAB1 was concluded as anti-inflammatory and cathepsin C inhibiting agent and could be used in the drug development against respective diseases.

## Graphical abstract

**Figure:**
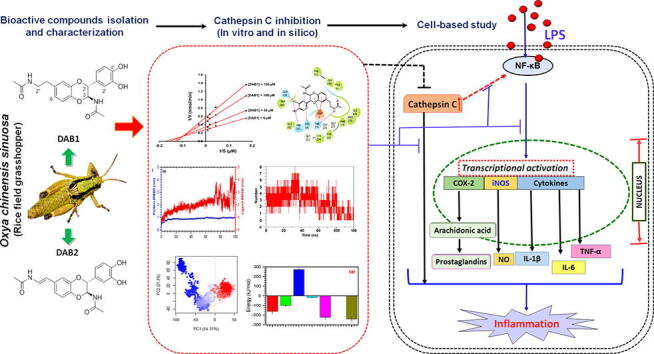

## 1 Introduction

Inflammation is an important innate immunity first-line defensive barrier stimulated by various physical and chemical agents, tissue injury, invading microorganism, and endotoxin [1]. During inflammation, immune cells like macrophages, neutrophils, and mast cells generate excessive amount of free radicals and a variety of pro-inflammatory cytokines as a defensive mediator [2]. However, persistently high inflammation leads to many severe pathological outcomes that include diabetes, cardiovascular disease, inflammatory bowel disease, rheumatoid arthritis and, various types of cancer [3], [4], [5]. Therefore, regulation of inflammation is mandatory to overcome the fatal effect exerted by inflammatory conditions.

Numerous transcriptional factors, such as activator of transcription and signal transducer, nuclear factor-kappa B (NF-κB), hypoxia-inducible factors, activation protein-1, and erythroid 2-related factor 2, are active during inflammation [5]. Besides, many studies suggested the involvement of cathepsins in the inflammation [6], [7]. Among the different groups of cathepsins, pivotal roles of cathepsin C have been reported in the induction and modulation of serine proteases and inflammatory responses [8], [9]. Likewise, a decreased inflammatory cell filtration and cytokine production in cathepsin C knockout mouse suggested an essential role of cathepsin in inflammation [10]. Thus, inhibition of cathepsin C could be a promising alternative approach to counter inflammatory disorders.

A number of natural products derived from animals and plants have been purported to have nutritional and medicinal values. In particular, plant-derived products have been studied, concerning various biological activities including anti-inflammatory activity [11] and cathepsin inhibitory potentials [12]. However, insects as a source of natural products have not been explored extensively; thus, provide huge possibilities as novel and effective agents against numerous diseases. The most common insect species utilized as food and medicine belong to orders Coleoptera, Lepidoptera, Hymenoptera, Orthoptera, and Hemiptera [13]. Among them, *Oxya chinensis sinuosa* (rice field grasshopper) which belongs to order Orthoptera of Acrididae family [14] is widely utilized in Korea and other Asian countries since millennia because of its high nutritional and medicinal value [15]. Traditionally, *O. chinensis sinuosa* is reported to cure several ailments including asthma, bronchitis, cough, paralysis, and seizures [16]. Knowing the many health benefits and safe to consume, recently Korea Food and Drug Administration has listed rice field grasshopper as a registered food for human intake [15]. Although, *O. chinensis sinuosa* is a common edible insect in Korea, still a little is known about the functional properties of its isolated compounds.

Therefore, this study examined the anti-inflammatory and cathepsin C inhibitory potential of compounds isolated from *O. chinensis sinuosa* using Raw 264.7 murine macrophage cells stimulated by lipopolysaccharide (LPS). Initially, bioactive compounds were isolated from the selected insect (*O. chinensis*), followed by their anti-inflammatory and *in vitro* cathepsin C inhibitory profiling. Furthermore, *in silico* approaches were used to provide insights into intermolecular interaction of the isolated bioactive compounds with cathepsin C.

## 2 Materials and methods

### 2.1 Chemical and reagents

All the chemical and reagents of analytical grade were used in the study. Antibody for inducible nitric oxide synthases (iNOS) was obtained from Abcam (Cambridge, MA, USA). Also, antibodies for cyclooxygenase-2 (COX-2), nuclear factor of kappa light polypeptide gene enhancer in B cells inhibitor alpha (IκBα), nuclear factor kappa B (NF-κB), interleukin 6 (IL-6), and β-actin were bought from Santa Cruz Biotechnology (Santa Cruz, CA, USA). The active form of mouse cathepsin C was procured from R&D Systems (Minneapolis, MN, USA).

### 2.2 Insect material

*O*. *chinensis sinuosa* samples were provided by the National Academy of Agricultural Science, Rural Development Administration, Wanju, Jeonbuk, Republic of Korea. A sample (CNUINS 201603) has been preserved as specimen in the archive of Pharmacognosy Laboratory of Chungnam National University (Daejeon, Republic of Korea).

#### 2.2.1 Extraction and isolation

Two *N*-acetyldopamine dimers, i.e. (2R,3S)-2-(3′,4′-dihydroxyphenyl)-3-acetylamino-7-(*N*-acetyl-2″-aminoethyl)-1,4-benzodioxane (DAB1) and (2R,3S)-2-(3′,4′-dihydroxyphenyl)-3-acetylamino-7-(*N*-acetyl-2″-aminethylene)-1,4-benzodioxane (DAB2), as bioactive compounds were isolated and purified from *O. chinensis sinuosa* [15]. Briefly, an ethanol (EtOH) extract (421.8 g) of the insect material was initially separated by silica gel VLC eluting with a gradient of n-hexane/ethyl acetate (EtOAc) (1:0, 0.8:0.2, 0.6:0.4, 0.4:0.6, 0.2:0.8) and a gradient of chloroform/methanol CHCl_3_/MeOH (1:0, 0.8:0.2, 0.6:0.4, 0.4:0.6, 0.2:0.8, 0:1) to produce six fractions of A-F. Fraction D (35.7 g) was further separated using silica gel vacuum liquid chromatography using a gradient mobile system of n-hexane/EtOAc (1:0, 0.85:0.15, 0.75:0.25, 0.5:0.5) and CHCl_3_/MeOH (0.85:0.15, 0.8:0.2, 0.67:0.33, 0.5:0.5, 0:1) to yield 6 sub-fractions of D1-D6. Fraction D5 (18.3 g) was chromatographed on reversed phase medium pressure liquid chromatography using a SNAP Cartridge KP-C18-HS, (340 g) and eluted with a gradient solvent system acetonitrile (MeCN)/H_2_O (0.1:0.9 → 0.6:0.4) to afford six fractions of D5-1-D5-6. Compound 1 (389.2 mg) and compound 2 (37.2 mg) were isolated from fraction D5-2 (1.9 g) by preparative high-performance liquid chromatography using a Phenomenex Kinetex C18 column (250 mm × 21.2 mm, 5 μm) and a mobile phase of MeCN/H_2_O (0.2:0.8 → 0.25:0.75)].

### 2.3 Cells and cell culture

Murine macrophage cell line RAW 264.7 was procured from American Type Culture Collection (ATCC, Manassas, VA, USA) and cultured in Dulbecco^’^s Modified Eagles Medium (Invitrogen, Carlsbad, CA, USA) supplemented with 10% fetal calf serum (Invitrogen), and 1% penicillin–streptomycin with an atmosphere of 5% CO_2_ at 37 °C incubator.

### 2.4 Cell viability assay

Effects of DAB1 and DAB2 on the viability of RAW 264.7 cells were examined using the 3-(4,5-dimethylthiazol-2-yl)-2,5-diphenyltetrazolium bromide (MTT) assay. Briefly, 1 × 10^4^ cells/well were seeded in a 96-well culture plate and incubated for 24 h at 37 °C in a 5% CO_2_ incubator. Cells were further treated with DAB1 or DAB2 (0–500 µM) for 24 h, followed by the addition of MTT dye (5 mg/ml) for 4 h. Finally, the formed formazan crystals were re-dissolved in dimethyl sulfoxide, and sample absorbance was recorded at 540 nm.

### 2.5 Determination of nitric oxide (NO)

NO was quantified calorimetrically using the Griess reagent. RAW 264.7 cells (1 × 10^5^ cells/well) were grown in a 6-well culture plate for 24 h and treated with DAB1 or DAB2 (0–250 µM) for 1 h, followed by the supplement of LPS (1 μg/ml) for 24 h. Finally, the cell-free supernatant was collected and processed to quantify NO by mixing 150 µl of cell-free supernatant with 100 µl of Griess reagent. After 30 min of incubation, absorbance at 540 nm was recorded and results were expressed as NO%.

### 2.6 Immunoblot analysis

RAW 264.7 cells were grown in 6-well culture plate (1 × 10^5^ cells/well) for 24 h. Cells were treated with DAB1 (250 µM) for 1 h, followed by exposure to LPS (1 µg/ml) for 12 h. After treatment, protein lysate was obtained using RIPA lysis buffer (Sigma-Aldrich, St. Louis, MO, USA). Finally, protein content was quantified and equal amount of protein (30 µg) was loaded in SDS-PAGE (10%) and processed for Western blots, as described by Khaket et al. [17].

### 2.7 Subcellular fractionation

The NE-PER nuclear protein extraction kit (Thermo Scientific, Rockford, USA) was used as per the manufacturer's instructions for obtaining subcellular fractions (cytoplasmic and nuclear).

### 2.8 Estimation of cytokine and prostaglandin E2

RAW 264.7 cells (1 × 10^5^ cells/well) were grown in a 6-well culture plate and treated with DAB1 (0–250 µM) for 1 h, followed by the treatment of LPS (1 μg/ml) for 12 h. Cell free culture media was collected and centrifuged at 10,000×*g* for 5 min. Supernatant was collected and processed for the quantification of TNF-α and IL-1β using ELISA kit (Invitrogen, Frederick, MD, USA) according to manufacture instructions. Prostaglandin E2 (PGE2) was quantified using a commercial competitive ELISA kit (Cayman Chemical, Michigan, USA) according to the manufacturer’s instructions.

### 2.9 Cellular cathepsin C activity

RAW 264.7 cells were grown in a 24-well culture plate (1 × 10^4^ cells/well) and then treated with DAB1 (0, 100, and 250 µM) together with LPS (1 µg/ml) for 12 h. Finally, cells were washed with phosphate buffered saline and lysed using CelLytic™ M Reagent (Sigma-Aldrich, St. Louis, MO, USA). The cell lysate was centrifuged at 10,000×*g* for 5 min in cooling conditions. The supernatant was collected and used as a source of lysate extract (crude enzyme). Enzyme activity was determined colorimetrically as previously described [17]. Briefly, lysate extract (10 µl) was mixed with 75 µl of assay buffer (50 mM CH_3_COONa, 10 mM NaCl, 1 mM DTT, and 1 mM EDTA, pH 5.5) and incubated for 10 min at 37 °C. Subsequently, 40 µM H-Gly-Phe-β naphthylamide was added. After 30 min incubation at 37 °C, 100 µl of sodium acetate buffer and 50 µl of coupling regent (0.1% Fast Garnet GBC in water) were added. The pink colored product was separated from the reaction mixture by partitioning with butanol, and the absorbance at 520 nm was recorded. The specific activity of cathepsin C was determined and expressed as percentage specific activity, considering only LPS treated control as 100%.

### 2.10 Cathepsin C inhibition assay

Enzyme inhibition activity was determined by mixing different concentrations of DAB1 (50–250 µM) with cathepsin C (10 ng/ml). The mixture was incubated at 37 °C for 15 min, followed by the addition of 40 µM substrate H-Gly-Phe-β naphthylamide. After 30 min incubation at 37 °C, 100 µl of sodium acetate buffer and 50 µl of coupling regent (0.1% Fast Garnet GBC in water) were added in the reaction mixture. The formed pink colored product was extracted with butanol and absorbance at 520 nm was recorded. Simultaneously, a control without inhibitor (DAB1) was prepared under the similar experimental conditions. Results were expressed as percent enzymatic activity, considering control as 100%.

### 2.11 Assessment of inhibition type and inhibition constant

Mode of inhibition was determined by caring out the enzyme reaction in presence and absence of inhibitor (DAB1) with varying amount of substrate. The enzyme cathepsin C (10 ng/ml) was incubated for 15 min with different concentrations of inhibitor (0, 50, 100, and 150 µM), followed by the addition of varied amount of substrate (10–100 μM) in assay buffer. After 30 min incubation at 37 °C, the enzymatic activity at each substrate concentration was determined. Finally, a double reciprocal Lineweaver-Burk plot was constructed against substrate concentration and velocity to determine Michaleis-Menten constant (K_m_), maximal velocity (V_max_), and inhibition type. Additionally, Eadie-Hofstee plot was also constructed for the cathepsin C DAB1 inhibition by plotting a graph between velocity and velocity/substrate concentrations.

### 2.12 In silico study

#### 2.12.1 Receptor and ligand structure

Three-dimensional structure of cathepsin C (PDB ID: 3PDF) [18] was retrieved from the Protein Data Bank (http://www.rcsb.org), and ligand (DAB1) three-dimensional structure was designed in the academic version of Maestro v12.3 (Schrödinger Release 2020–1:Maestro, Schrödinger, LLC, New York, NY, 2020). The co-crystallized cyanamide-based inhibitor compound 17 binding region [18] was selected as an active pocket in the cathepsin crystal structure for the molecular docking simulation with DAB1 compound.

#### 2.12.2 Molecular docking

The molecular docking simulation for cathepsin C and compound DAB1 was performed using Chimera-AutoDock Vina plugin setup [19]. Briefly, both receptor and ligand structures were prepared, including addition of polar hydrogen and charges to each structure, for docking using Dock prep tool in Chimera-1.14 [20]. The selected receptor protein (cathepsin C) was the monomer unit consisting of a single heavy chain, light chain and the exclusion unit. The docking was conducted under default parameters in the selected active pocket of receptor covered by docking grid box of size 17.07 × 14.29 × 17.68 Å along the x, y, and z axes and center at 31.05, 26.05, and 18.57 Å region using AutoDock Vina [21], as a plugin in USCF Chimera-1.14. Finally, docked conformations were selected based on the highest docking score with the least root mean square deviation (RMSD) values and further analyzed for intermolecular interaction profile using Maestro v12.3 (Schrödinger). The docking studies were performed was performed on the active site of monomer

#### 2.12.3 Molecular dynamics simulation

To validate the stability and to monitor the intermolecular interaction profiling with respect to time for the generated conformation of cathepsin C docked with potent ligand, molecular dynamics simulation was carried out for 100 ns using the Groningen Machine for Chemicals Simulations (GROMACS) 2018.1 package. The topology of the receptor was generated by using the Charmm 27 force field [22] and the three-point transferable intermolecular potential water conditions. Swiss PARAM sever was used to generate the ligand topology file [23]. To resemble the physiological conditions, the simulation was performed at 0.15 M salt concentration balanced by the addition of Na^+^ or Cl^−^ ions. Later, system energy minimization was performed through nsteps = 50,000 through steepest descent method (1000 ps) and conjugated descent approach (100 ps). Moreover, the Particle Mesh Ewald approach was employed to calculate the energy as well as intermolecular interactions including van der Waals interactions. While a 14 Å distance limit was fixed for the short-range radius of van der Waals along with Coulomb cut-off and neighbor list were adjusted at a distance of 9 Å [24]. Besides, the linear constraint solver algorithm was used to compute the covalent bond constraints [25], and a time step of 0.002 ps was fixed to produce molecular dynamic simulation trajectory. Finally, 100 ns molecular dynamics simulation was performed for each docked complex with nsteps of size 500,000,000. Furthermore, the generated trajectories were studied for three intrinsic properties to analyze the complex stability, i.e. RMSD, root mean square fluctuation (RMSF), and the frequency of hydrogen bonds formation with respect to 100 ns interval using GROMACS utilities.

#### 2.12.4 Principal components and dynamic cross-correlation matrix analysis

The essential dynamics, in terms of principal component analysis (PCA), which reflect the essential motions in protein required for enzymatic activity and residual displacement in the docked complex during molecular dynamic simulation with respect to time were calculated using Bio3d package [26], as reported by Bharadwaj et al. [27].

#### 2.12.5 Binding free energy calculation

The major energy components responsible for cathepsin C-DAB1 complex stability were computed by the molecular mechanics Poisson-Boltzmann surface area (MM/PBSA) method, as described by Kumari et al. [28] using Adaptive Poisson-Boltzmann Solver [29] to resolve the Possion-Boltzman equations. The complete calculations were conducted as per given instructions in tutorial of g_MM/PBSA tool at http://rashmikumari.github.io/g_mmpbsa/accessed.21040920 [28]. The end-point binding free energy calculated from MM/PBSA was further utilized to predict the theoretical Ki value using the equation, Ki = Δbinding energy/R × T, where R is the gas constant (1.985 × 10^−3^ kcal/mol) and T represent temperature (298.15 K).

### 2.13 Statistical analysis

All the experiments were performed in triplicates, and results are presented as mean ± SD. SPSS-16 software was used to determine the statically difference between the groups employing one way ANOVA using the Duncan test for post hoc analysis at *p*-value < 0.05.

## 3 Results and discussion

### 3.1 Identification of isolated compounds

Compound 1 (DAB1): yellowish amorphous powder;${\left[a\right]}_{D}^{23}$ −36.2, (MeOH, c 0.1); 1H nuclear magnetic resonance [NMR] (600 MHz, MeOH‑*d*_4_) *δ* 6.84–6.73 (6H, overlap, H-5, H-6, H-8, H-2‘, H-5′, H-6′), 5.67 (1H, d, *J* = 7.11 Hz, H-3), 4.69 (1H, d, *J* = 7.11 Hz, H-2), 3.35 (2H, t, *J* = 7.16 Hz, H-2″), 2.69 (2H, t, *J* = 7.16 Hz, H-1″), 1.90 (3H, s, CH3), 1.87 (3H, s, CH3); 13C NMR (150 MHz) *δ* 173.3 (CO), 173.2 (CO), 147.1 (C-4′), 146.5 (C-3′), 144.3 (C-8a), 142.2 (C-4a), 134.2 (C-7), 128.8 (C-1′), 123.2 (C-6), 120.6 (C-6′), 118.1 (C-8), 117.9 (C-5), 116.1 (C-5′), 115.6 (C-2′), 78.3 (C-2), 78.3 (C-3), 42.1 (C-2″), 35.8 (C-1″), 22.6 (CH3), 22.5 (CH3); electrospray ionization-mass spectrometry [ESI-MS] *m*/*z* 387 [M + H]+

Compound 2 (DAB2): yellowish amorphous powder;${\left[a\right]}_{D}^{23}$ −31.4, (MeOH, c 0.1); 1H NMR (600 MHz, MeOH‑*d*_4_) *δ* 7.30 (1H, d, *J* = 14.68 Hz, H-2″), 6.90 (1H, d, *J* = 1.71 Hz, H-2′), 6.86 (1H, dd, *J* = 1.93, 8.28 Hz, H-6), 6.85 (1H, brs, H-5′), 6.81 (1H, d, *J* = 1.93 Hz, H-8), 6.76 (1H, d, *J* = 8.28 Hz, H-5), 6.74 (1H, dd, *J* = 1.71, 8.14 Hz, H-6′), 6.10 (1H, d, *J* = 14.68 Hz, H-1″), 5.69 (1H, d, *J* = 7.13 Hz, H-3), 4.71 (1H, d, *J* = 7.13 Hz, H-2), 2.03 (3H, s, CH3), 1.87 (3H, s, CH3), 13C NMR (150 MHz) *δ* 173.2 (CO), 170.6 (CO), 147.2 (C-4′), 146.5 (C-3′), 144.6 (C-8a), 142.6 (C-4a), 131.9 (C-7), 128.7 (C-1′), 122.9 (C-2″), 120.6 (C-6′), 120.3 (C-6), 118.3 (C-8), 116.1 (C-5), 115.6 (C-5′), 114.7 (C-2′), 114.0 (C-1″), 78.4 (C-2), 78.3 (C-3), 22.6 (CH3), 22.6 (CH3); ESIMS *m*/*z* 407 [M + Na]+.

### 3.2 Structure determination of isolated compounds

**Fig. 1:**
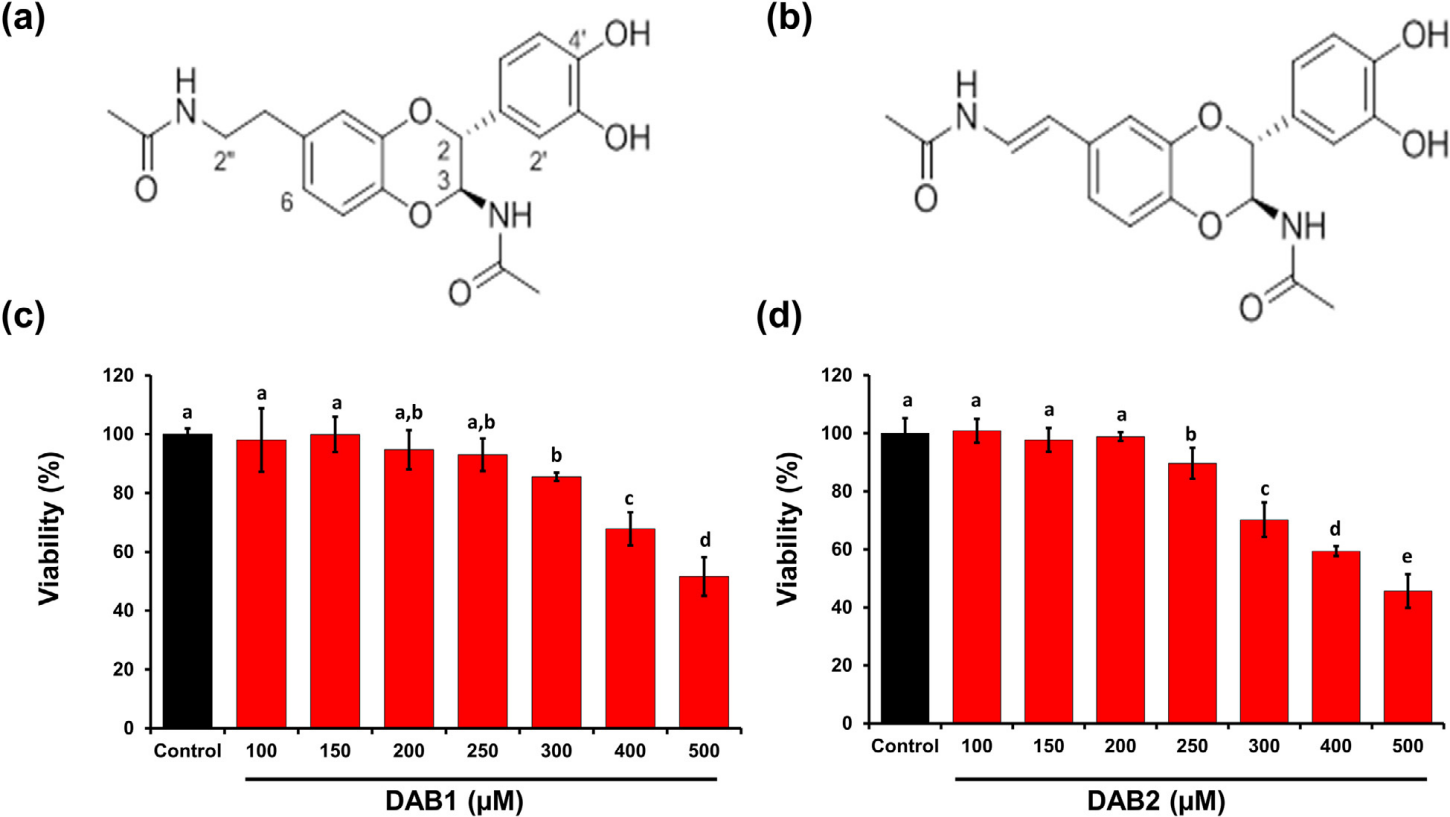
Chemical structures of two *N*-acetyldopamine dimers, **(a)** (2R,3S)-2-(3′,4′-dihydroxyphenyl)-3-acetylamino-7-(*N*-acetyl-2″-aminoethyl)-1,4-benzodioxane (DAB1) and **(b)** (2R,3S)-2-(3′,4′-dihydroxyphenyl)-3-acetylamino-7-(*N*-acetyl-2″-aminethylene)-1,4-benzodioxane (DAB2) from *O. chinensis sinuosa* and their effect on the viability of Raw 264.7 murine macrophages evaluated by MTT assay. **(c)** & (**d)** Cell viabilities in the presence of DAB1 and DAB2 (100–500 µM), respectively. Each value represents the mean ± standard deviation. In bar graphs letters with different alphabets represent the significant difference at *p* < 0.05, based on Duncan^,^s multiple comparison test.

The structures of isolated bioactive compounds, i.e. DAB1 and DAB2, were identified using mass spectroscopy (MS) and nuclear magnetic resonance (NMR) techniques (Fig. S1-S8). The NMR spectroscopic data of DAB1 displayed characteristic signals for a typical *N*-acetyldopamine derivative. Six aromatic protons at 6.84–6.73, two methines at 5.67 (1H, d, *J* = 7.11 Hz, H-3), 4.69 (1H, d, *J* = 7.11 Hz, H-2), two methylenes at 3.35 (2H, t, *J* = 7.16 Hz, H-2″) and 2.69 (2H, t, *J* = 7.16 Hz, H-1″), and two acetyl groups at 1.90 (3H, s) and 1.87 (3H, s) were observed in the ^1^H NMR spectrum. The 13C NMR spectroscopic data revealed 20 carbon resonances assigned to two carbonyl groups (δC 173.3, 173.2), twelve aromatic carbons (δC 147.1, 146.5, 144.3, 142.2, 134.2, 128.8, 123.2, 120.6, 118.1, 117.9, 116.1, 115.6), two methines (δC 78.3, 78.3), two methylenes (δC 42.1, 35.8), and two acetyl groups (δC 22.6, 22.5). The planar structure was determined to be 2-(3′,4′-dihydroxyphenyl)-3-acetylamino-7-(*N*-acetyl-2″-aminoethyl)-1,4-benzodioxane based on NMR spectroscopy analysis. The coupling constant between H-2 and H-3 was 7.11 Hz, indicating that it possesses *trans* H-2/H-3 stereochemistry. To determine the absolute stereochemistry, circular dichroism (CD) analysis was performed (Fig. S4-S8). The absolute configurations at C-2 and C-3 were confirmed to be 2R and 3S by comparison of the experimental CD spectrum with those in the literature [15]. The structure of compound 1 (DAB1) was identified as (2R,3S)-2-(3′,4′-dihydroxyphenyl)-3-acetylamino-7-(*N*-acetyl-2″-aminoethyl)-1,4-benzodioxane (Fig. 1a).

The ^1^H NMR and ^13^C NMR spectra of compound 2 (DAB2) were similar to those of compound 1 (DAB1), except two additional olefinic protons (δH 7.30, 6.10) and the absence of proton signals for the two methylene groups. The absolute configuration was determined to be 2R, 3S, based on the same Cotton effect as that of DAB1. Consequently, the structure of DAB2 was identified as (2R,3S)-2-(3′,4′-dihydroxyphenyl)-3-acetylamino-7-(*N*-acetyl-2″-aminethylene)-1,4-benzodioxane (Fig. 1b).

### 3.3 Cell viability assay

Results of cell viability in the presence of DAB1 and DAB2 are depicted in Fig. 1c and Fig. 1d. DAB1 showed non-significant changes in the cell viability up to 250 µM. However, beyond this concentration cell viability was reduced significantly by DAB1 and DAB2. Therefore, a concentration up to 250 µM was selected for further studies. At concentrations beyond 250 µM, DAB2 was more cytotoxic than DAB1 (Fig. 1c and Fig. 1d). At 300 µM, cell viability of DAB1 and DAB2 treated samples was 85.53 ± 1.37% and 70.20 ± 5.98%, respectively. There is limited literature available on the isolation and biological activity evaluation of the compounds isolated from *O. chinensis sinuosa* [15]. However, a few studies were carried out in the *O. chinensis sinuosa* extract, elucidating their effect on the macrophage [30] and HepG2 cells viability [31].

### 3.4 Effect on NO production

**Fig. 2:**
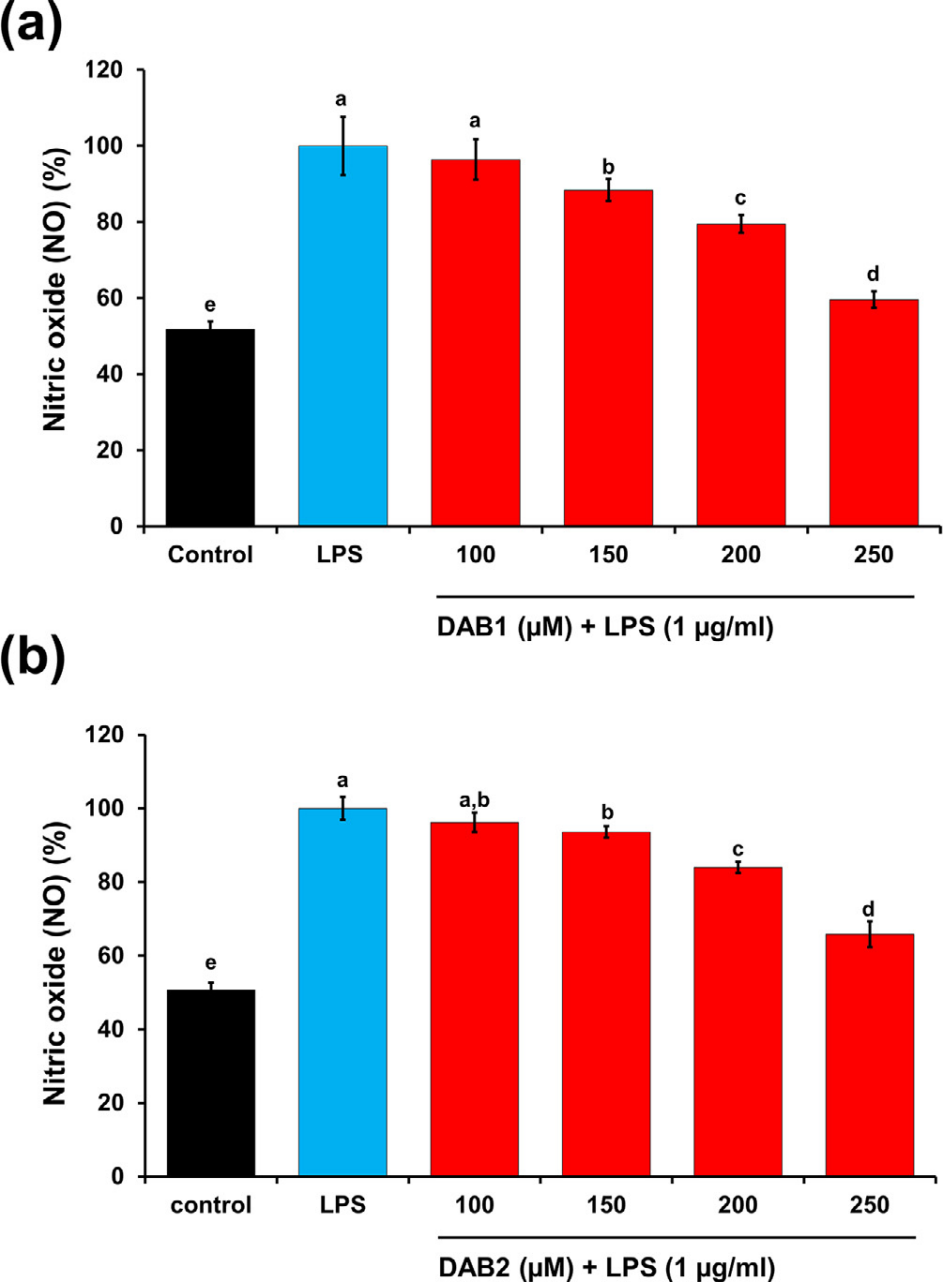
Effect of compounds **(a)** DAB1 and **(b)** DAB2 (100–250 µM) on NO production in LPS (1 µg/ml) stimulated Raw 264.7 macrophages. Each value represents the mean ± standard deviation. In bar graphs letters with different alphabets represent the significant difference at *p* < 0.05, based on Duncan’s multiple comparison test.

The vigorous involvement of NO in many inflammatory disorders has been documented [32]. Given this background, effect of DAB1 and DAB2 on NO generation in LPS stimulated macrophages was also studied. DAB1 and DAB2 reduced the production of NO in LPS stimulated cells dose-dependently (Fig. 2). At 250 µM, DAB1 and DAB2 treated macrophages displayed NO levels of 59.56 ± 2.12% and 65.79 ± 3.50%, respectively, as compared to 100% in macrophages stimulated only with LPS (Fig. 2). DAB1, which reduced NO more levels than DAB2, was selected for subsequent experiments to examine other inflammatory targets.

### 3.5 Expression of iNOS and COX-2

**Fig. 3:**
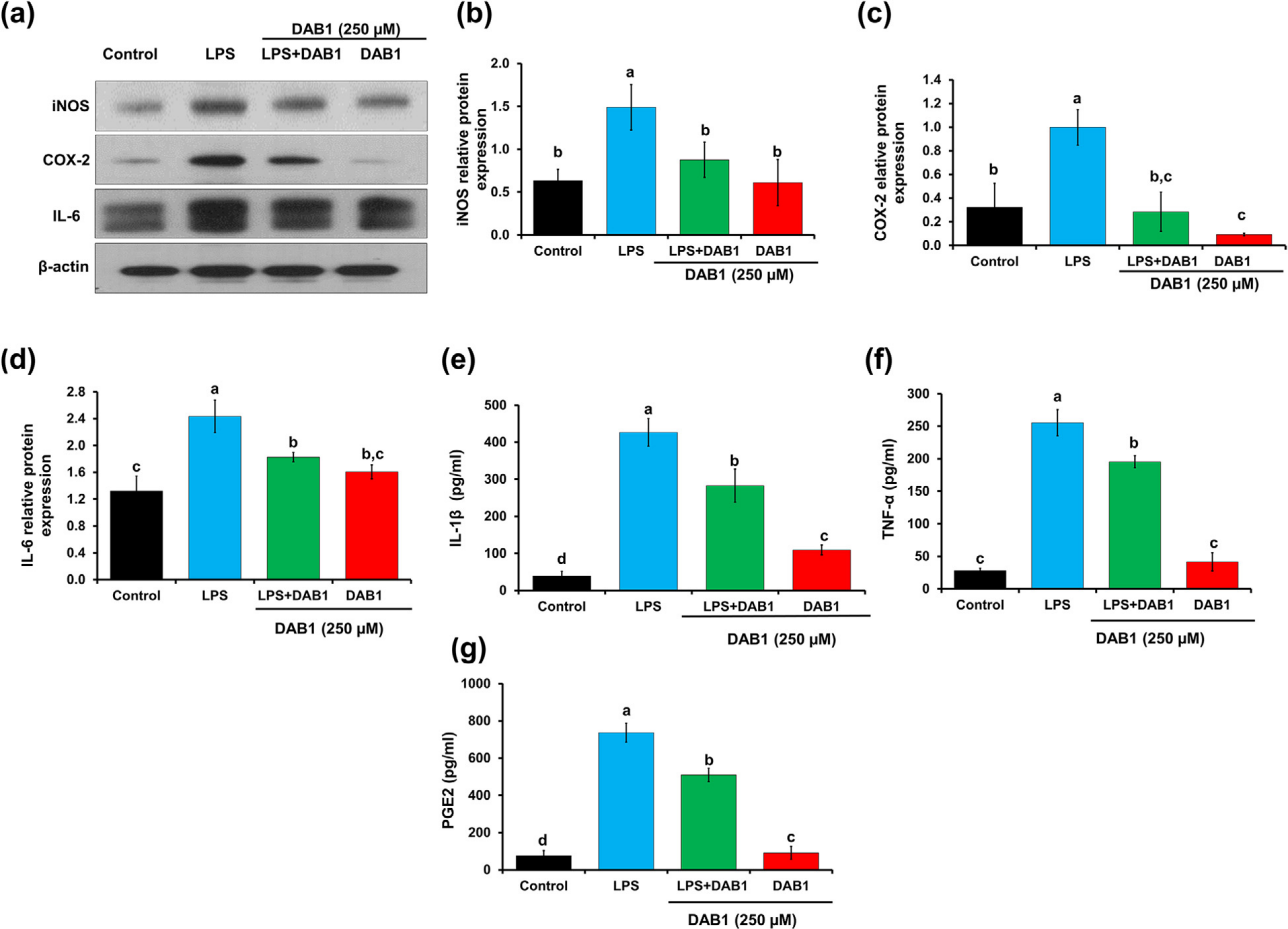
Effect of DAB1 on the inflammatory markers and cytokines in the LPS stimulated Raw 264.7 murine macrophages. β-actin was used as a loading control. **(a)** Western blotting for iNOS, COX-2, and IL-6. **(b)**, **(c)**, and **(d)** are the densitometry analyses of iNOS, COX-2, and IL-6, respectively. (**e)** Quantification of secretory IL-1β, **(f)** quantification of secretory TNF-α, **(g)** quantification of PGE2. Each value represents the mean ± standard deviation. In bar graphs letters with different alphabets represent the significant difference at *p* < 0.05, based on Duncan^,^s multiple comparison test.

Inflammatory pathogenesis almost always features the higher expression of iNOS and its associated NO [33]. At 250 µM, DAB1 significantly reduced the expression of iNOS in LPS stimulated cells (*p* < 0.05) (Fig. 3a and Fig. 3b). A 1.7-fold lower iNOS expression was detected in LPS stimulated cells treated with DAB1, as compared to only LPS treated cells, signifying anti-inflammatory potential of DAB1 (Fig. 3a and b).

Together with iNOS, higher expression of COX-2 has also been documented in various inflammatory diseases [34], [35]. As depicted in Fig. 3, DAB1 effectively reduced the LPS induced expression of COX-2. A 3.5-fold lower COX-2 expression was noticed in the LPS stimulated cells treated with DAB1, as compared to only LPS treated cells (Fig. 3a and c).

So far, no literature suggests direct evidence of compounds isolated from *O. chinensis sinuosa* against iNOS and COX-2 expression. However, the study done by Yoon et al. [30] revealed the effect of *O. chinensis sinuosa* extract on iNOS and COX-2 expression. It is conceivable that the crude extract contained DAB1 as one of the major components responsible for downregulation of iNOS and COX-2.

### 3.6 Effect of DAB1 on the production of cytokine and prostaglandin E2

A positive correlation between augmentation of cytokines, including IL-6, IL-1β, TNF-α, and inflammatory disorders have been established [36]. A significant contribution of IL-6 has been observed in inflammation [37], [38]. A massive production of IL-6 was evident in the cells treated with LPS, compared to untreated control cells (Fig. 3a and d). In contrast, cells treated with DAB1 significantly reduced LPS augmented IL-6 expression. A significant 1.3-fold reduction in the level of IL-6 was observed in macrophages treated with DAB1 and LPS, as compared to cells treated only with LPS (Fig. 3d). The critical role of IL-1β has been observed in many pathological conditions, and its reduced production can be beneficial in inflammatory disorders [39]. Treatment of LPS resulted in the massive production of IL-1β, compared to untreated control cells (Fig. 3e). However, production of IL-1β was substantially reduced in LPS induced macrophages treated with DAB1. A significantly (*p* < 0.05) 1.5-fold reduced production of IL-1β was observed in macrophages treated with DAB1 and LPS, as compared to cells treated only with LPS (Fig. 3e). The active involvement of TNF-α has been confirmed in several inflammatory disorders, such as ankylosing spondylitis and Crohn's disease [5]. As expected, DAB1 effectively reduced TNF-α production in LPS stimulated cells. A significantly (*p* < 0.05) 1.3-fold lower production of TNF-α was observed in macrophages treated with DAB1 and LPS, compared to only LPS stimulated macrophages (Fig. 3f).

COX-2 is highly upregulated by various stimuli including LPS and leads to the production of PGE2 which have a critical role in inflammation and pain [40]. Herein PGE2 level was determined by the ELISA assay which revealed a significantly (*p* < 0.05) 1.4-fold reduction of PGE2 level in the macrophages treated with DAB1 prior to the LPS compared to only LPS treated cells (Fig. 3g).

There is no direct evidence to indicate the effect of compounds isolated from *O. chinensis sinuosa* on cytokine expression. As mentioned above, Yoon et al. [30] reported the effect of ethanol extract of *O. chinensis sinuosa* on the expression on TNF-α, IL-6, and IL-1β in LPS stimulated macrophages. Additionally, many insect extracts and dopamine derivatives isolated from these extracts were effective in preventing the inhibition of these important cytokines [41].

### 3.7 Repression of NF-κB signaling pathway

**Fig. 4:**
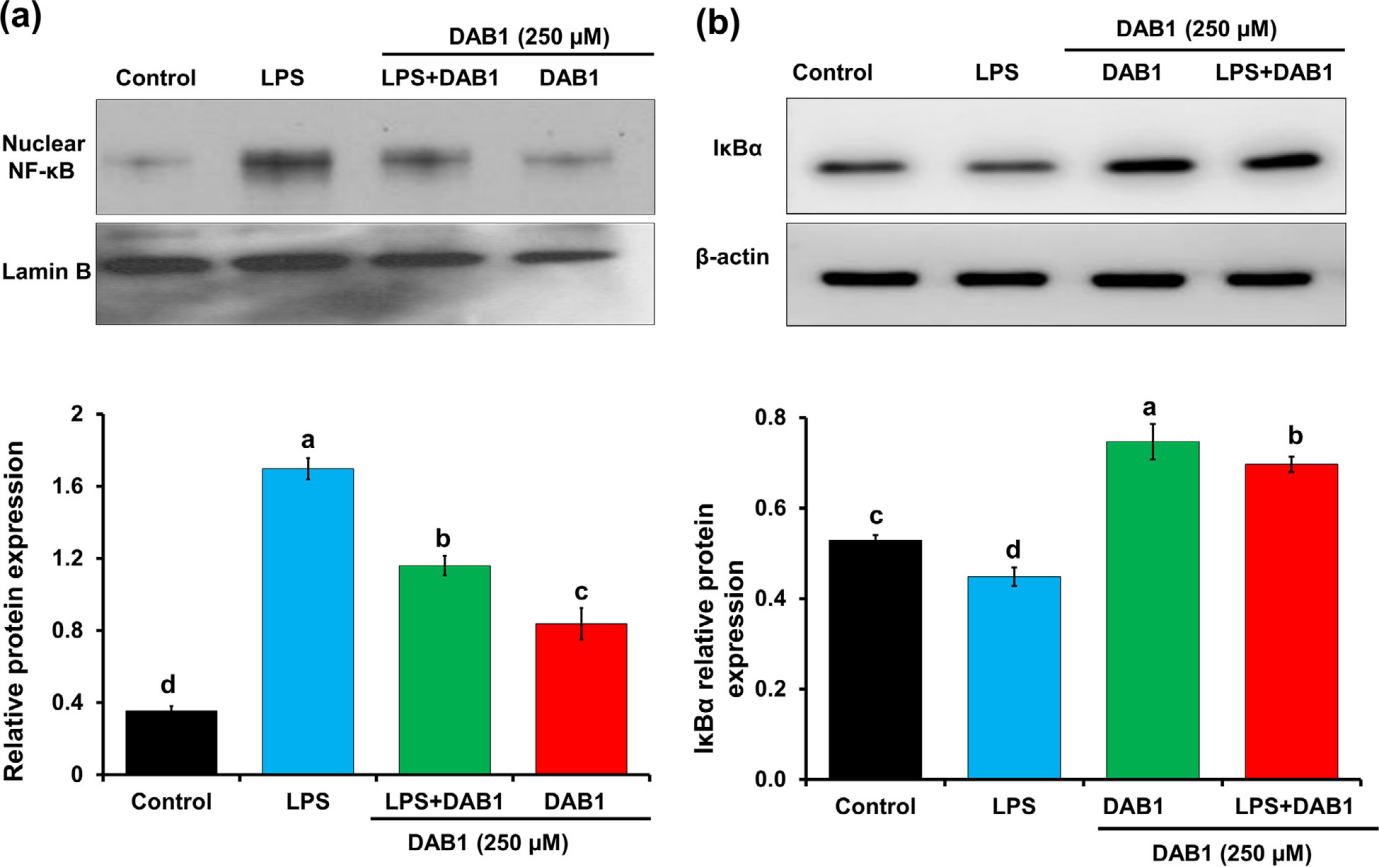
Effect of DAB1 in the **(a)** nuclear translocation of NF-κB and **(b)** IκBα degradation in LPS stimulated Raw 264.7 murine macrophages. Tubulin and β-actin were used as loading controls. In bar graphs letters with different alphabets represent the significant difference at *p* < 0.05.

NF-κB is the most viable pharmacological target against inflammation, reflecting its vital regulatory role in the expression of important inflammatory proteins [5]. Few reports showed that cathepsin C promotes neuroinflammation via activation of Ca^+2^ dependent PKC/p38MAPK/NF-κB pathway [10]. Knowing the key importance of NF-κB in the regulation of inflammation, we examined the efficacy of DAB1 on NF-κB translocation by immunoblotting. Translocation of NF-κB was more pronounced in LPS stimulated cells (Fig. 4a). However, reduced translocation was observed in LPS stimulated cells treated with DAB1. A significantly (*p* < 0.05) 1.5-fold lower nuclear translocation of NF-κB was observed in macrophages treated with LPS and DAB1, as compared to macrophages stimulated with LPS, suggesting a preventive role of DAB1 on NF-κB translocation and eventually inflammation (Fig. 4a). During the physiological condition, NF-κB is attached with inhibitor of NF-κB, IκBα, and degradation of IκBα is essential for the activation and translocation of NF-κB [42]. We assessed the role of DAB1 on the degradation of IκBα. As shown in Fig. 4b, LPS enhanced the degradation of IκBα with 1.12-fold, as compared to control. However, in the cells treated with DAB1, the LPS induced degradation of IκBα was essentially reversed.

The results collectively suggest the anti-inflammatory behavior of DAB1 that involves the inhibition of NF-κB translocation mediated by IκBα degradation. Although the effect of *O. chinensis sinuosa* extract on the translocation of NF-κB has been described [30], no prior study has addressed the effect of isolated compounds from *O. chinensis sinuosa* on the translocation of NF-κB.

### 3.8 Cellular cathepsin C activity

**Fig. 5:**
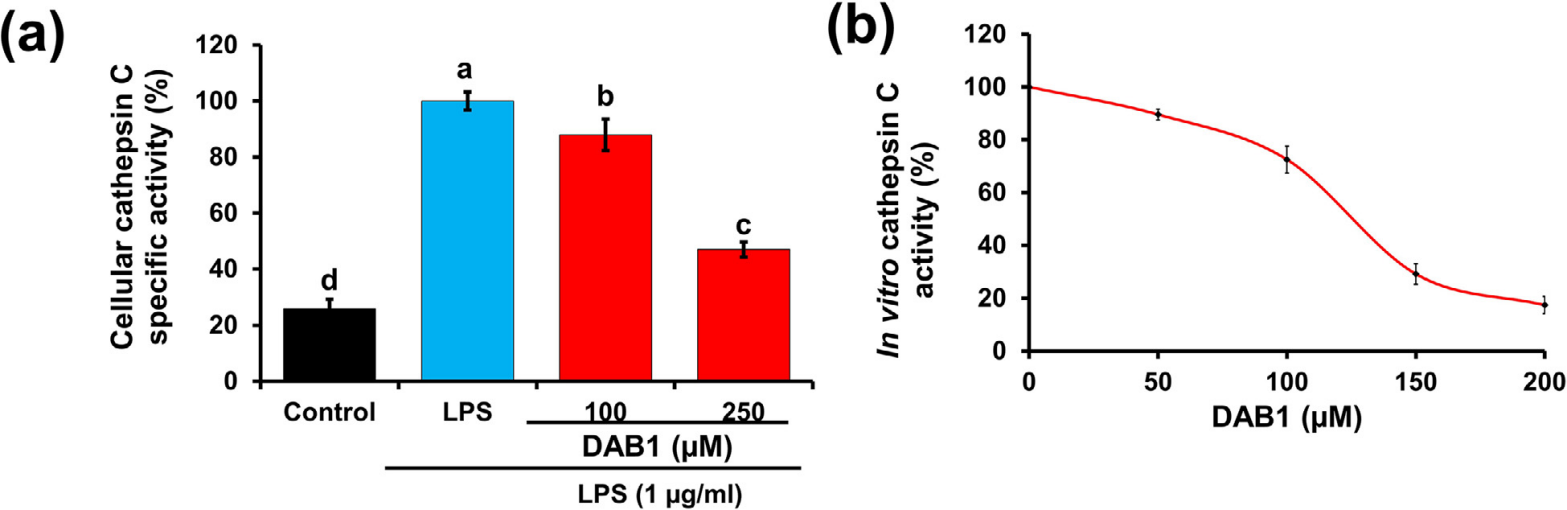
Effect of DAB1 on the cellular and *in vitro* cathepsin C activity. **(a)** Cathepsin C activity in Raw 264.7 murine macrophages in the absence and presence of DAB1. **(b)***In vitro* cathepsin C inhibition by DAB1 with purified enzyme. Each value represents the mean ± standard deviation. In bar graphs letters with different alphabets represent the significant difference at *p* < 0.05.

Various clinical and preclinical studies have highlighted the involvement of cathepsin C in inflammation [43]. Active participation of cathepsin C has been verified in the expression of iNOS and IL-1β and in the translocation of NF-κB [8]. Given the noteworthy role of cathepsin C in inflammation, we examined the effect of DAB1 on the cathepsin C activity. LPS induced cells displayed higher cathepsin C activity, which was subsequently inhibited by DAB1 in a dose-dependent manner. Cathepsin C activity in cells treated with 100 and 250 µM DAB1 was 87.93 ± 5.63% and 46.98 ± 2.69%, respectively, compared to 100% in cell stimulated only with LPS (Fig. 5a). The findings provided elementary evidence that cathepsin C might be a possible target of DAB1, which is responsible for its anti-inflammatory behavior. This idea is supported by the demonstrated involvement of cathepsin C to stimulate FAK/PIK-2, an upstream target of IκBα and NF-κB in macrophages [8]. We speculated that DAB1 might be modulating the anti-inflammatory activity via inhibition of cathepsin C protein. However, a detailed study is required to establish and confirm this notion.

### 3.9 In vitro cathepsin C inhibition assay

**Fig. 6:**
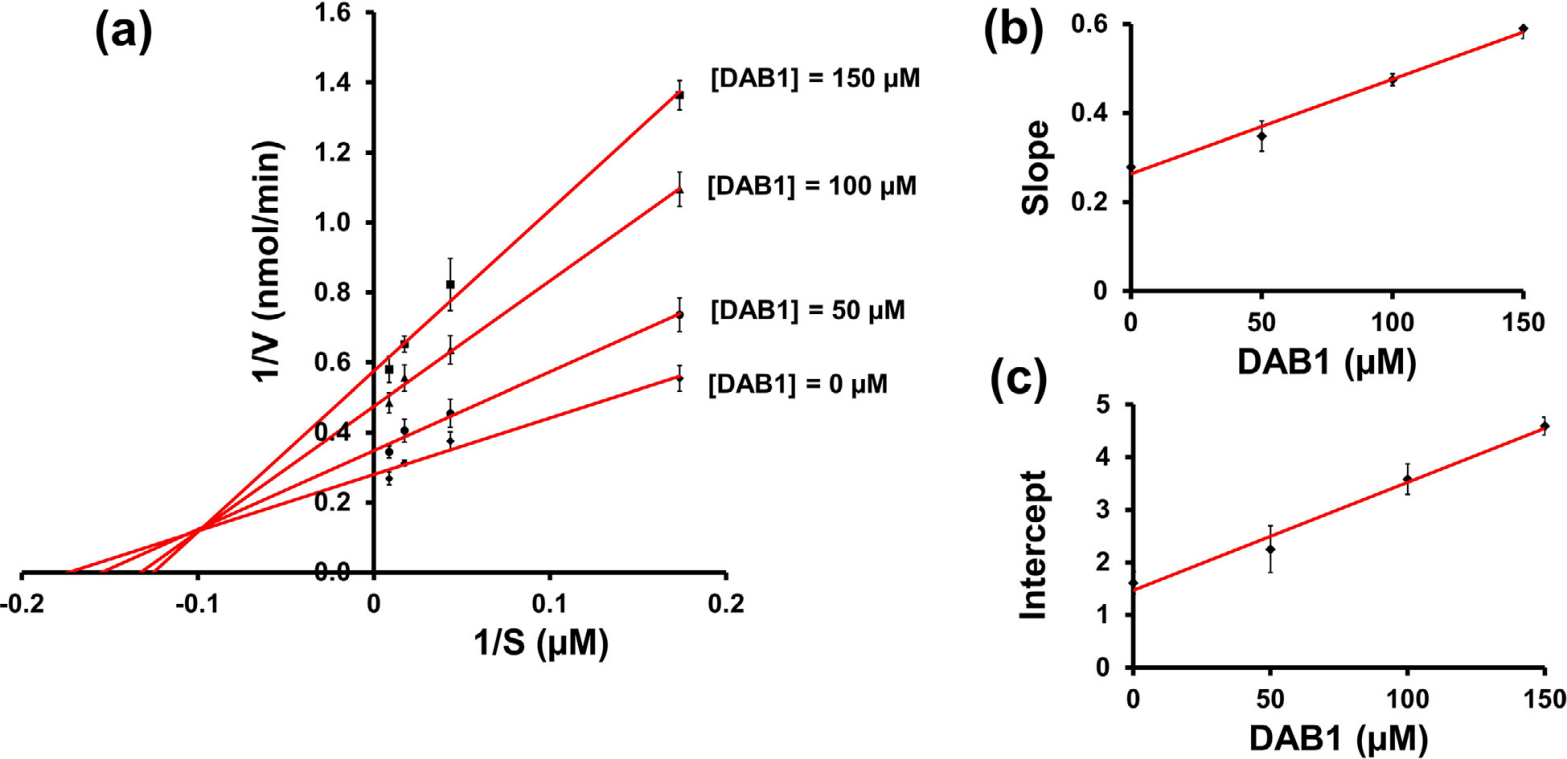
Cathepsin C inhibition kinetics by DAB1. **(a)** Lineweaver-Burk plots. **(b)** The secondary plot between slope and different concentrations of DAB1 for the determination of inhibition constant (K_i_) **(c)** The plot of intercept versus the DAB1 concentrations for the determination of inhibition constant (K_is_).

The cellular cathepsin C assay reported decreased activity of cathepsin C in the cells treated with DAB1. Decreased activity might be due to the effect of DAB1 on cathepsin C expression or by the direct interaction with an expressed product (cathepsin C), followed by blockage of the activity. To discern between the two possibilities, an *in vitro* inhibitory assay involving DAB1 and pure cathepsin C was performed. The enzyme inhibition assay revealed that DAB1 effectively inhibited cathepsin C with an inhibitory concentration fifty (IC_50_) of 126.03 ± 5.04 µM (Fig. 5b). The inhibitory kinetic behavior was assessed using the Lineweaver-Burk plot and revealed that the line originating from uninhibited and inhibited enzymes merged in the left side of the 1/V axis with reducing K_m_ and rising V_max_. The results indicated a mixed-type inhibition (Fig. 6). The K_m_ value of the uninhibited enzyme was 5.75 ± 0.75 µM, which was 1.32-fold (7.57 ± 0.62 µM) and 1.35-fold (7.76 ± 0.14 µM) lower, compared to the K_m_ value obtained using 100 and 150 µM DAB1, respectively. The equilibrium constant of the inhibitor for the free enzyme (inhibition constant, K_i_, 71.56 ± 10.21 µM) and enzyme-substrate complex (K_is_, 133.55 ± 18.2 µM) was derived from a secondary plot between inhibitor vs. slope and inhibitor vs. intercept, respectively (Fig. 6b and Fig. 6c). The inhibition constant values of DAB1 towards cathepsin C were slightly high that limiting its application as a pharmaceutical agent. However, it can be compensated owing to the presence of DAB1 in the edible food product (rice field grasshopper) that promotes its consumption as a functional food. Additionally, DAB1 can be served as a lead compound to derive more promising compounds as a cathepsin C inhibitor. Inhibition kinetics was also determined by the Eadie-Hofstee plot that supports the findings obtained from the Lineweaver-Burk plot (Fig. S9). The K_m_ value of the uninhabited enzyme was 6.14 µM which was 1.25-fold (7.69 µM) and 1.34-fold (8.29 µM) higher than the K_m_ value observed for the enzyme treated with 100 and 150 µM of DAB1, respectively. The finding of the Eadie-Hofstee plot are comparable with the results obtained from the Lineweaver-Burk plot. These findings provided confirmatory evidence that DAB1 can inhibit cathepsin C. To the best of our knowledge, cathepsin C inhibition by isolate bioactive compounds *O. chinensis sinuosa* and its extract were not reported in the literature. Therefore, the current findings indicate the potential of DAB1 as a novel inhibitor that could be effective against cathepsin C mediated ailments, including inflammation.

### 3.10 Molecular docking and dynamics simulation

**Fig. 7:**
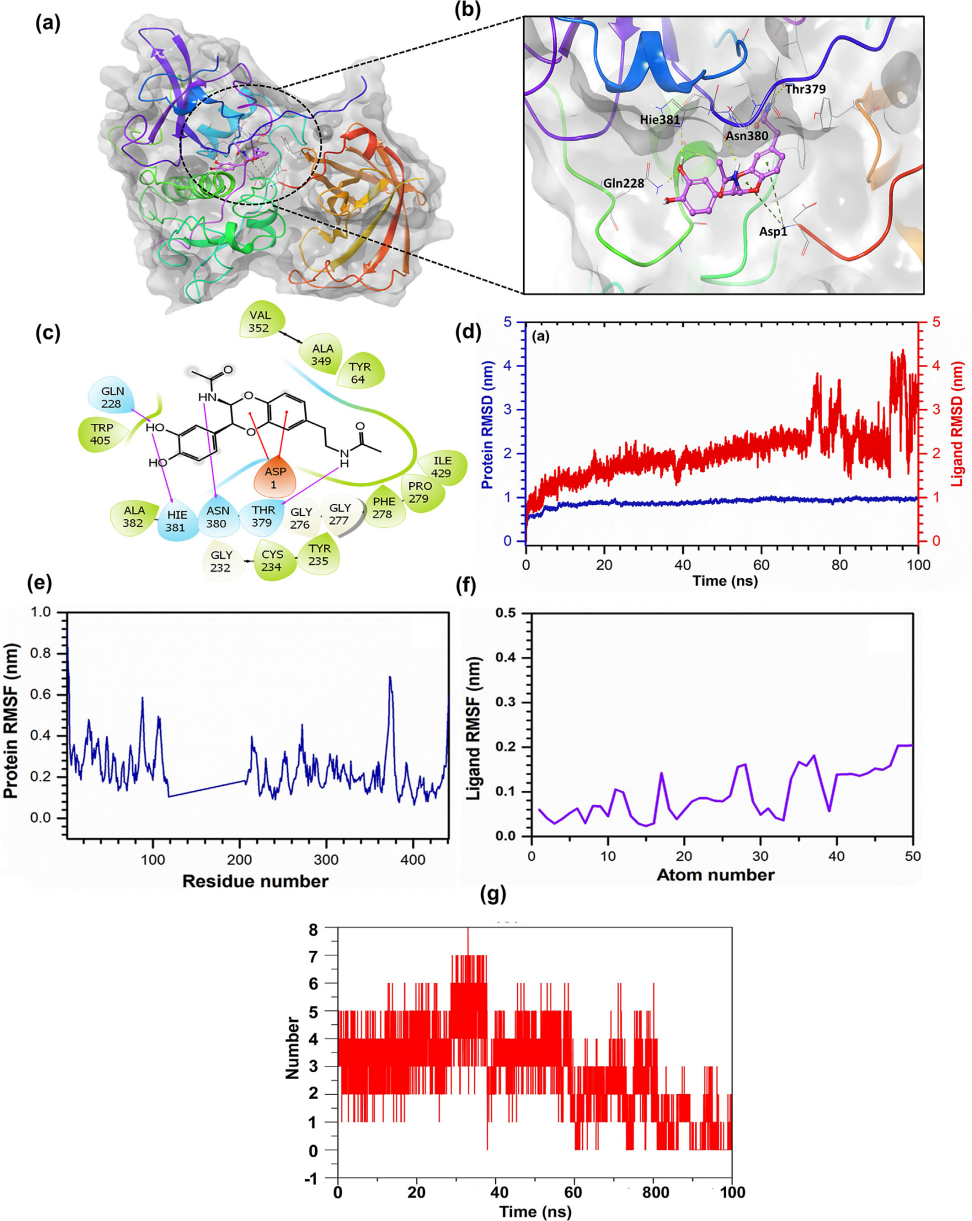
*In silico* interaction of DAB1 with cathepsin C. **(a)** and **(b)** 3D structure of the docked complex of cathepsin C-DAB1. **(c)** 2D mapping of the cathepsin C-DAB1 complex monitored within 4 Å space around the ligand in the active pocket of cathepsin C. Pink color arrows represent the hydrogen bonds, while green, blue, and grey color residues stand for the hydrophobic, polar, and glycine interactions between cathepsin C and DAB1. **(d)** RMSD value was extracted from the 100 ns molecular dynamics simulation trajectories for cathepsin C alpha carbon atom and ligand DAB1. **(e)** and **(f)** RMSF plot was generated for the cathepsin C-DAB1 docked complex and ligand DAB1, respectively. **(g)** Hydrogen-bond formation between protein and ligand as function of time.

To extend and validate the *in vitro* inhibitory results, molecular docking was performed (Fig. 7a-c). As *in vitro* results revealed the DAB1 interaction with the active site of the cathepsin C (Fig. 6), therefore, docking was primarily focused on the active pocket of monomeric cathepsin C made by S1 unit of heavy chain and S2 unit of light chain. Docking results revealed a substantial binding affinity (-8.72 kcal/mol) of DAB1 with cathepsin C by the formation of four strong hydrogen bonds with residues Gln228, Thr379, Asn380, and Hie381 (histidine neutral ε protonated) (Fig. 7b-c). Besides, the docked complex was effectively stabilized by the involvement of ten hydrophobic interactions at Tyr64, Cys234, Tyr235, Phe278, Pro279, Val352, Ala349, Ile429, Ala382, and Trp405 residues of the cathepsin C with DAB1 (Fig. 7c**).** Importantly DAB1 showed the interaction with important amino acid residues (Asp1, Cys234, Hie381) which together form a catalytic ternary complex [44]. Additionally, negative, polar, and glycine interactions were also logged in the docked complexes of cathepsin C with DAB1 (Fig. 7c). These interacting residues were also reported in the crystal structure of cathepsin C [18], suggesting that DAB1 has the potential to inhibit cathepsin C, as observed from the *in vitro* data (Fig. 5, Fig. 6).

### 3.11 Stability and interaction map analysis

The stability of the docked DAB1 with cathepsin C was evaluated using 100 ns molecular dynamic simulation. The RMSD values (<1 nm) noted for the receptor suggested stability of the protein (Fig. 7d). This is further supported by acceptable RMSF values (<0.6 nm) of the residues in the protein, except in the region of interaction with the ligand DAB1 (<0.3) (Fig. 7e). Moreover, RMSF values recorded for the protein fit ligand were also computed from 100 ns molecular dynamic simulation trajectory and suggested considerable RMSD values at <3.6 nm with variations till the end of the simulation, indicating that complex required more simulation interval to achieve the equilibrium (Fig. 7d). Also, calculated RMSF values for the ligand further supported the stability of the ligand in the active pocket of cathepsin C (<0.2 nm) (Fig. 7f). Furthermore, the computation of the hydrogen bonds with their maximum magnitude at 8 and a minimum of 3 number between the cathepsin C and DAB1 during molecular dynamic simulations demonstrated the stability of the respective docked complex (Fig. 7g). Overall, the outcome of both RMSD and RMSF values signifies the stability of cathepsin C-DAB1 complex.

### 3.12 PCA and DCCM analysis

**Fig. 8:**
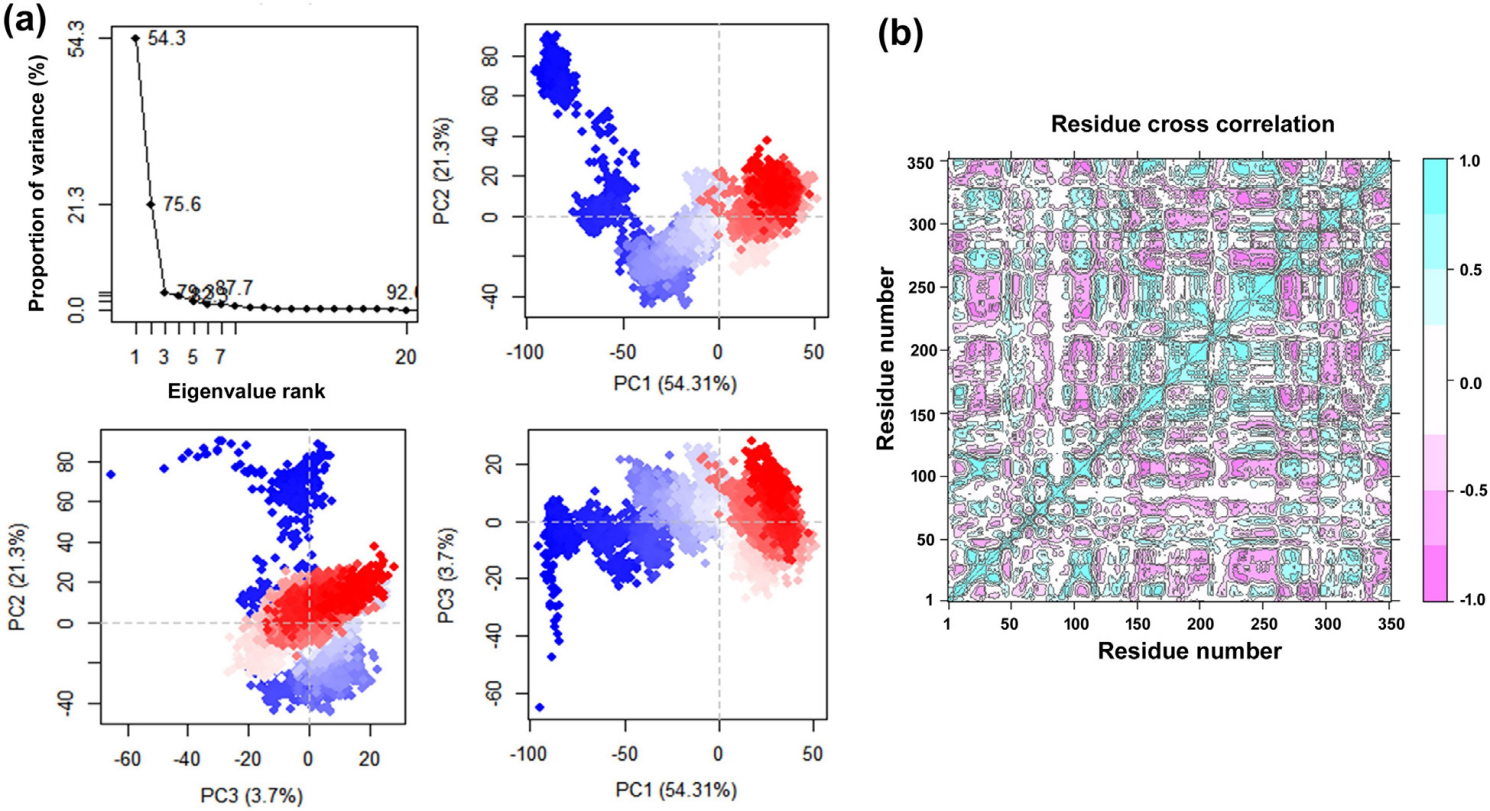
PCA, and DCCM analysis for the molecular dynamics simulation trajectories of cathepsin C-DAB1 complex. **(a)** PCA, the percentage of overall mean square displacement of residue positional fluctuation noted in each dimension is expressed by corresponding eigen value (PCs). The color values blue to white to red representing the periodic jump during 100 ns simulation. **(b)** Dynamic cross correlation for cathepsin C docked with DAB1. The positive and negative correlation in the movement of residue is represented as cyan and red color, respectively.

PCA was used to determine the domain dynamics motion in the docked receptor with ligand during 100 ns simulation and represented as fraction of variance (eigen fraction) with covariance matrix at 20 eigen model. The receptor cathepsin C is the monomer unit consisting of a single heavy chain, light chain and the exclusion unit. The DAB1 docked on the active pocket made by S1 unit of heavy chain and S2 unit of light chain. As depicted in Fig. 8a, a sudden drop of 79.8% variance at first three eigen modes, which indicates a significant conformational change in cathepsin C active pocket (S1 and S2 unit) induced by DAB1. Later the value reached to the static elbow point and showed no considerable variations in the calculated eigen fraction following 4 till 20 eigen values (Fig. 8a). These variations in the complex during simulation interval are discussed as influence of DAB1 by interacting with the active residues which ultimately contributed to the protein–ligand complex stability. Fig. 8a represents the initial three PCA of eigen fraction as the cluster group obtained from the molecular dynamics simulation trajectory and supported by the initial flexibility in the complex followed by the state of compact cluster motions for cathepsin C structure with an increase in simulation interval. The 2D plots indicate the overall variation in cathepsin C conformation during simulation and the color shifting from red to blue showing gradual shift in structure during conformation changes.

Furthermore, the DAB1 induced conformational dynamic changes in cathepsin C was evaluated by DCCM analysis emphasizing on correlated motions during simulation at alpha carbon atom (Fig. 8b). The DCCM results revealed a substantial alteration in correlated motions and dynamics changes in structure of cathepsin C docked with DAB1 and are consistent with the findings of PCA plots.

### 3.13 Binding affinity evaluation

Table 1 show the endpoint binding free energy for the cathepsin C-DAB1 complex along with contributing energy components. The different energy values were computed by MM/PBSA method, as reported by Kumari et al. [28]. Herein, polar solvation energy was observed for positive contribution while electrostatic interactions, non-polar solvation energy, and van der Waals interaction energy values were noted to reduce the total free binding energy [45]. These findings suggested that van der Waals, electrostatic, and non-polar solvation energy values contribute to the stability of cathepsin C docked with DAB1. The binding energy of DAB1 with cathepsin C is -47.18 ± 3.48 kcal/mol (Table 1), indicating the considerable binding of DAB1 with cathepsin C. Furthermore, theoretical Ki value (79.72 ± 5.88 µM) was predicted for cathepsin C-DAB1 docked complex using end-point binding free energy calculated with MM/PBSA and found it to be comparable with the experimental Ki value (71.56 ± 10.21 µM) obtained from the *in vitro* assay. These results were coherent with the predictions of docking scores and the enzyme inhibition assay, suggested the bioactive compound DAB1 as an inhibitor of cathepsin C.

**Table 1:** MM/PBSA derived various energy components involved in the stabilization of cathepsin C-DAB1 complex.

Energy components	Energy (kcal/mol)
van der Waals energy (Δ_Vdw_)	−38.70 ± 4.33 kcal/mol
Electrostatic energy (Δ_Ele_)	−20.84 ± 1.66 kcal/mol
Polar solvation energy (Δ_Pol sol_)	43.20 ± 3.55 kcal/mol
Solvent accessible surface area energy (Δ_SASA_)	−4.38 ± 0.22 kcal/mol
Solvent accessible volume energy (Δ_SAV_)	−30.86 ± 3.61 kcal/mol
Weeks-Chandler-Andersen potential energy (Δ_WCA_)	0.00 ± 0.00 kcal/mol
Binding energy (Δ_BD_)	−47.18 ± 3.48 kcal/mol

## 4 Conclusion

DAB1 isolated from the *O. chinensis sinuosa* suppressed the expression of inflammatory mediators, iNOs and COX-2, including cytokines, via NF-κB signaling pathway in LPS induced macrophages. DAB1 effectively inhibited cathepsin C activity at the cellular level. Combined *in vitro* and *in silico* enzyme inhibition studies suggested a mixed-type inhibition. The cathepsin C inhibition and anti-inflammation behavior of DAB1 supports the notion of cathepsin C mediated anti-inflammatory activity via NF-κB signaling pathway. The collective results implicate DAB1 as a novel cathepsin C inhibitor that may be valuable as a preventive agent against inflammatory disorders after *in vivo* studies.

## CRediT authorship contribution statement

**Ashutosh Bahuguna:** Methodology, Writing – original draft. **Tejinder Pal Khaket:** Methodology, Writing – original draft. **Vivek K. Bajpai:** Software. **Shruti Shukla:** Software. **InWha Park:** Validation, Visualization. **MinKyun Na:** Validation, Visualization. **Yun Suk Huh:** Data curation. **Young-Kyu Han:** Data curation. **Sun Chul Kang:** Methodology. **Myunghee Kim:** Conceptualization, Supervision, Writing – review & editing.

## Declaration of Competing Interest

The authors declare that they have no known competing financial interests or personal relationships that could have appeared to influence the work reported in this paper.
