# Supplementary material for: N-Acetyldopamine dimers from Oxya chinensis sinuosa attenuates lipopolysaccharides induced inflammation and inhibits cathepsin C activity
Source: Comput Struct Biotechnol J. 2022 Feb 15;20:1177–88. doi: 10.1016/j.csbj.2022.02.011 (PMC8908036; doi:10.1016/j.csbj.2022.02.011)
Supplement: Supplementary data 1 [file mmc1.docx]

**Supplementary material**

***N*-acetyldopamine dimers from *Oxya chinensis sinuosa* attenuates lipopolysaccharides induced inflammation and inhibits cathepsin C activity**

Ashutosh Bahuguna^a,$^, Tejinder Pal Khaket^b$^, Vivek K. Bajpai^c,$^, Shruti Shukla^d^, InWha Park^e^, MinKyun Na^e,*^, Yun Suk Huh^f,*^, Young-Kyu Han^c,*^, Sun Chul Kang^b,*^, Myunghee Kim^a,g*^

^a^*Department of Food Science and Technology, Yeungnam University, Gyeongsan-si, Gyeongsangbuk-do 38541, Republic of Korea*

^b^*Department of Biotechnology, Daegu University, Gyeongsan-si, Gyeongsangbuk-do 78453, Republic of Korea*

^c^*Department of Energy and Materials Engineering, Dongguk University-Seoul, Seoul 04620, Republic of Korea*

^d^*Department of Food Science and Technology, National Institute of Food Technology Entrepreneurship and Management (NIFTEM), Sonipat, Haryana 131028, India*

^e^*College of Pharmacy, Chungnam National University, Daejeon 34134, Republic of Korea*

^f^*Department of Biological Engineering, NanoBio High-Tech Materials Research Center, Inha University, Incheon 22212, Republic of Korea*

*^g^Institute of Cell Culture, Yeungnam University, Gyeongsan-si, Gyeongsangbuk-do 38541, Republic of Korea*

^$^These authors equally contributed

**Corresponding authors:**

**Dr. MinKyun Na** ([mkna@cnu.ac.kr](mailto:mkna@cnu.ac.kr)); **Dr. Yun Suk Huh** ([yunsuk.huh@inha.ac.kr](mailto:yunsuk.huh@inha.ac.kr)); **Dr. Young-Kyu Han** ([ykenergy@dongguk.edu](mailto:ykenergy@dongguk.edu)); **Dr. Sun Chul Kang** ([sckang@daegu.ac.kr](mailto:sckang@daegu.ac.kr)); **Dr. Myunghee Kim** ([foodtech@ynu.ac.kr](mailto:foodtech@ynu.ac.kr)); Phone: +82-53-810-2958; Fax: +82-053-810-4662

Figure S1. ^1^H NMR of compound 1 (DAB1) (600 MHz, methanol-*d*_4_)

**Figure S2**. ^13^C NMR spectrum of compound **1 (DAB1)** (150 MHz, methanol-*d_4_*)


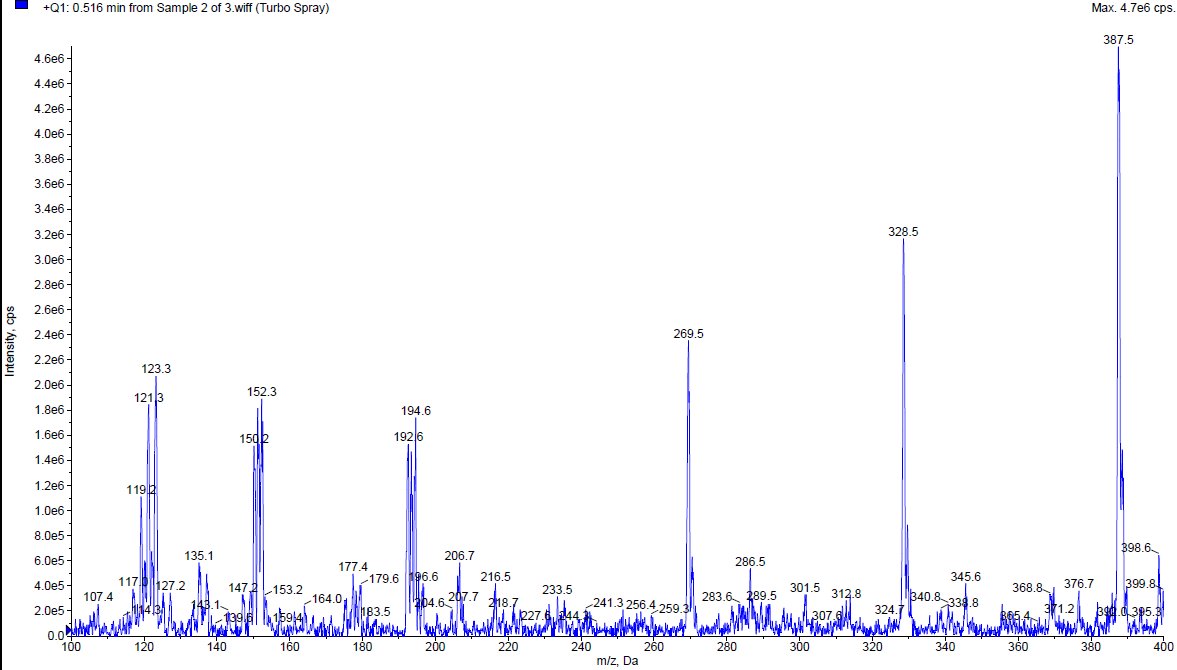


**Figure S3.** ^13^C NMR spectrum of compound **1 (DAB1)** (150 MHz, methanol-*d_4_*) ESIMS spectrum of compound **1 (DAB1)**


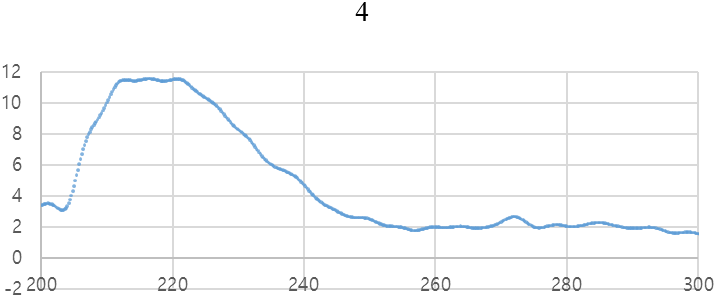


**Figure S4.** The CD spectrum of compound **1(DAB1)**

**Figure S5.** ^1^H NMR of compound **2 (DAB2)** (600 MHz, methanol-*d_4_*)

**Figure S6**. ^13^C NMR spectrum of compound **2 (DAB2)** (150 MHz, methanol-*d_4_*)


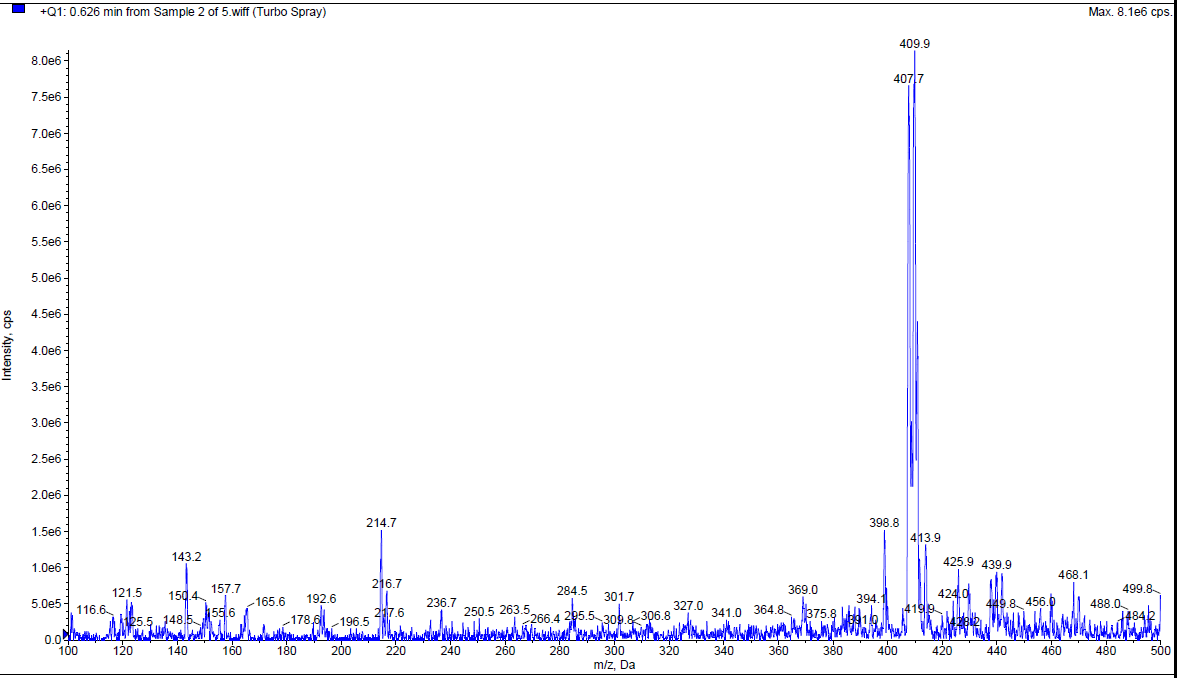


**Figure S7.** ESIMS spectrum of compound **2 (DAB2)**


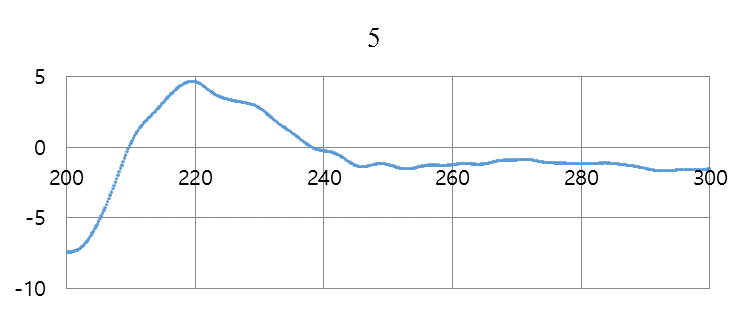


**Figure S8.** The CD spectrum of compound **2(DAB2)**

**Eadie–Hofstee plot**

Eadie–Hofstee plot was constructed against velocity of the reaction and function of the V/S. The Km and Vmax values of the uninhibited enzyme, enzyme inhibited with 50 µM, 100 µM and 150 µM of DAB1 was calculated using the question derived from the constructed Eadie–Hofstee plot using the equation:

$$Vo=-Km\frac{\mathrm{Vo}}{[S]}+Vmax$$

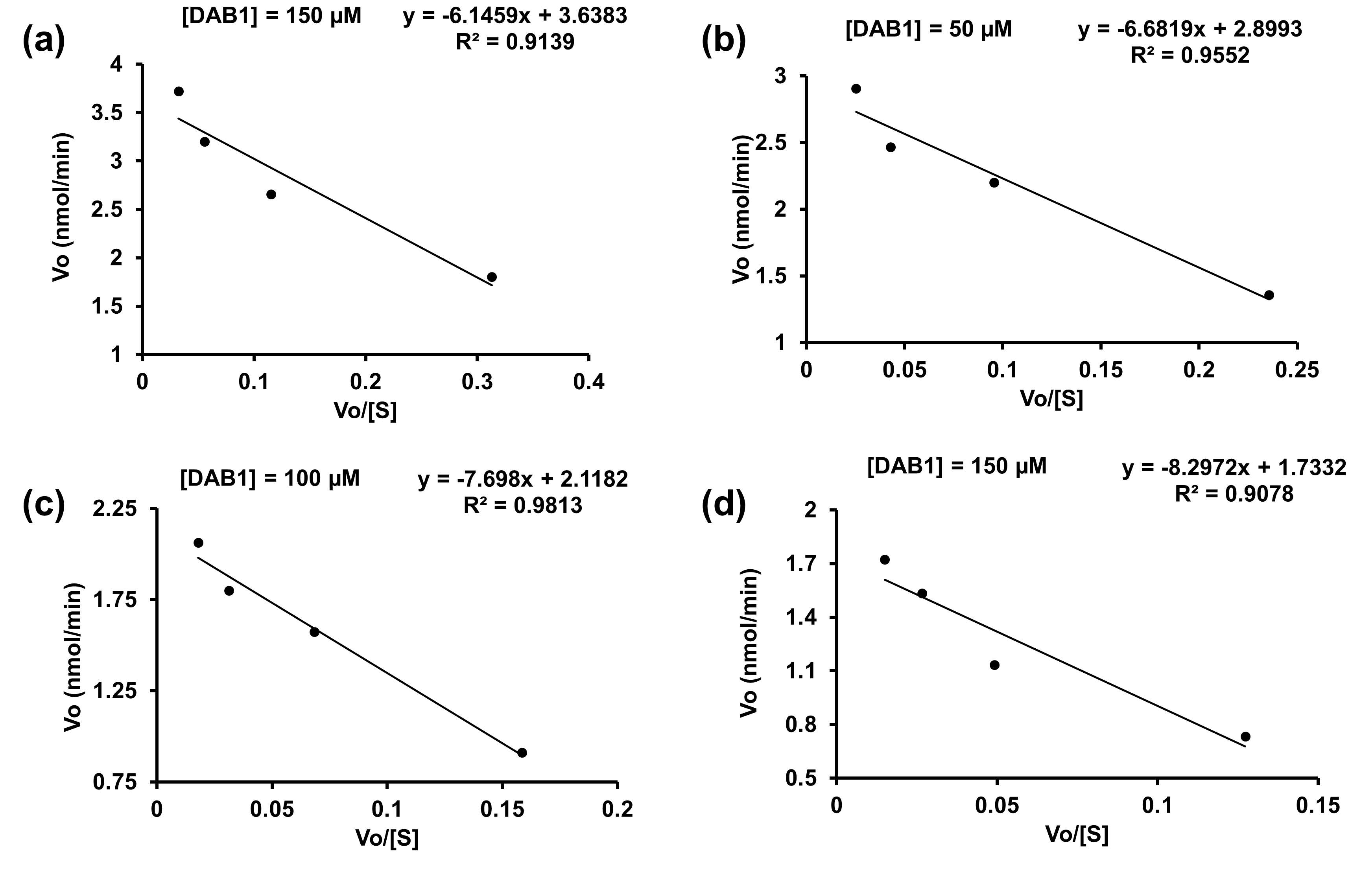


**Figure S9.** Cathepsin C inhibition kinetics by DAB1 derived from Eadie–Hofstee plot. (a) Without inhibitor (DAB1) (b) DAB1 50 μM (c) DAB1 100 μM (b) DAB1 150 μM.


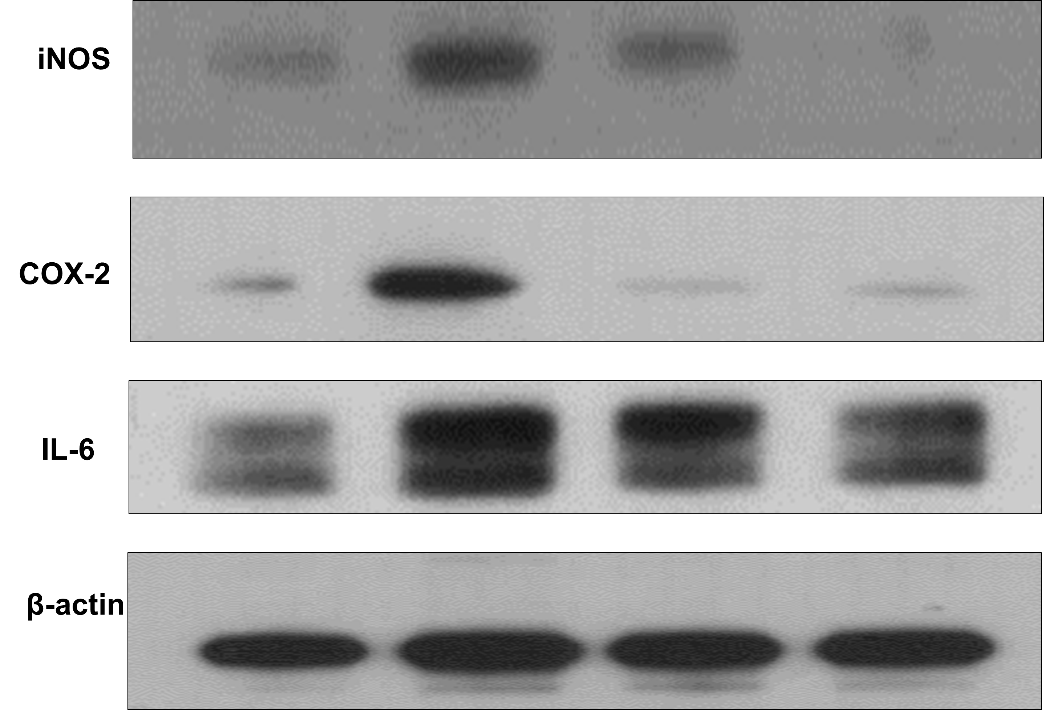


**Figure S10 a.** Western blots for for iNOS, COX-2, and IL-6


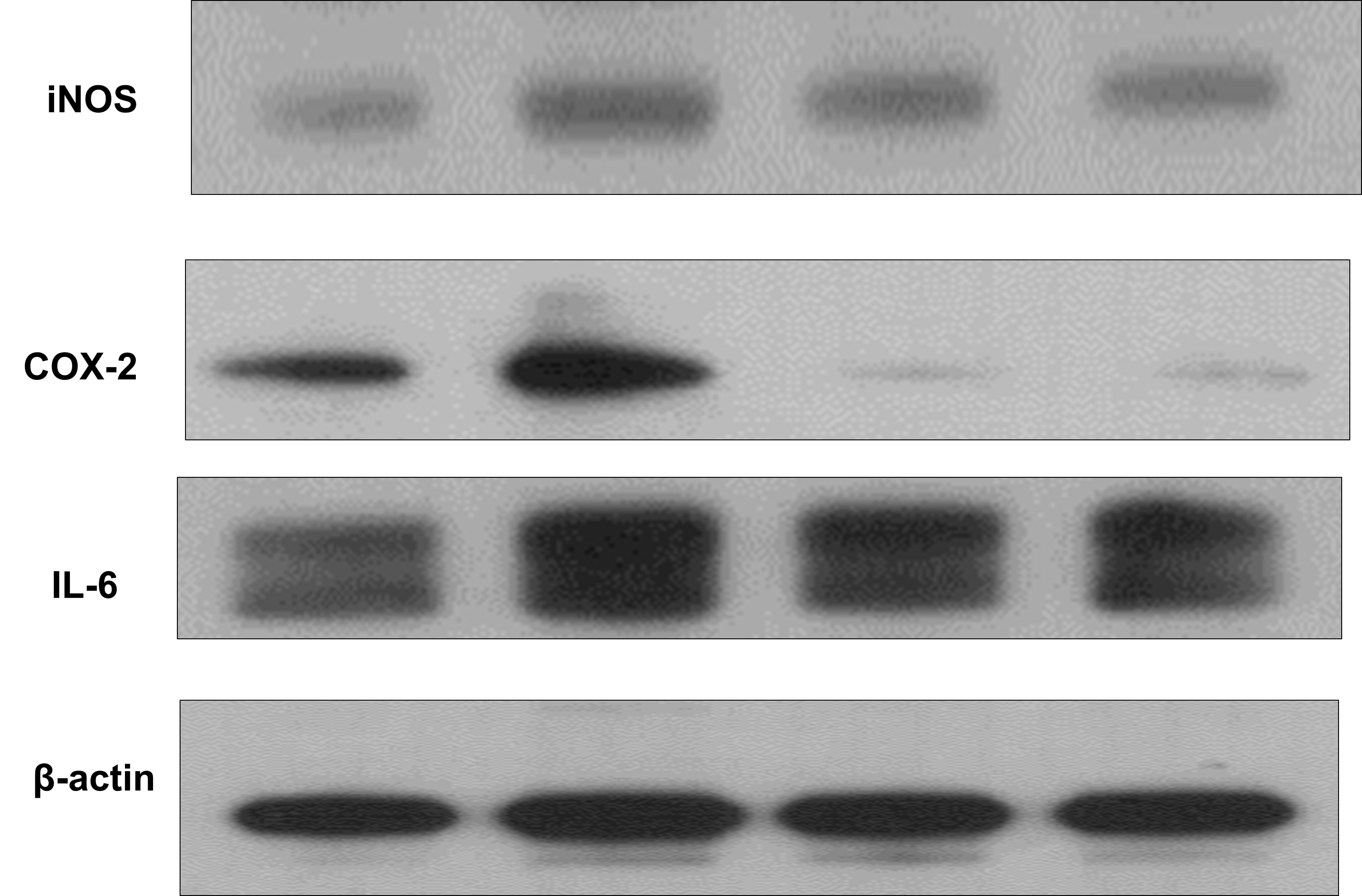


**Figure S10 b.** Western blots for for iNOS, COX-2, and IL-6
